# Proton vs. Photon Radiation Therapy for Primary Gliomas: An Analysis of the National Cancer Data Base

**DOI:** 10.3389/fonc.2018.00440

**Published:** 2018-11-28

**Authors:** Jaymin Jhaveri, En Cheng, Sibo Tian, Zachary Buchwald, Mudit Chowdhary, Yuan Liu, Theresa W. Gillespie, Jeffrey J. Olson, Aidnag Z. Diaz, Alfredo Voloschin, Bree R. Eaton, Ian R. Crocker, Mark W. McDonald, Walter J. Curran, Kirtesh R. Patel

**Affiliations:** ^1^Department of Radiation Oncology and Winship Cancer Institute, Emory University, Atlanta, GA, United States; ^2^Biostatistics and Bioinformatics Shared Resource, Winship Cancer Institute, Emory University, Atlanta, GA, United States; ^3^Department of Radiation Oncology, Rush University, Chicago, IL, United States; ^4^Department of Surgery, Emory University, Atlanta, GA, United States; ^5^Department of Neurosurgery and Winship Cancer Institute, Emory University, Atlanta, GA, United States; ^6^Department of Hematology and Medical Oncology and Winship Cancer Institute, Emory University, Atlanta, GA, United States; ^7^Department of Therapeutic Radiology, Yale University, New Haven, CT, United States

**Keywords:** proton therapy, gliomas, overall survival, IMRT, NCDB

## Abstract

**Background:** To investigate the impact of proton radiotherapy (PBT) on overall survival (OS) and evaluate PBT usage trends for patients with gliomas in the National Cancer Data Base (NCDB).

**Methods:** Patients with a diagnosis of World Health Organization (WHO) Grade I-IV glioma treated with definitive radiation therapy (RT) between the years of 2004–13 were identified. Patients were stratified based on WHO Grade and photon radiotherapy (XRT) vs. PBT. Univariate (UVA) and multivariable analysis (MVA) with OS were performed by Cox proportional hazards model and log-rank tests. Propensity score (PS) weighting was utilized to account for differences in patient characteristics and to minimize selection bias.

**Results:** There were a total of 49,405 patients treated with XRT and 170 patients treated with PBT. Median follow-up time was 62.1 months. On MVA, the following factors were associated with receipt of PBT (all *p* < 0.05): WHO Grade I-II gliomas, treatment at an academic/research program, west geographic facility location, and surgical resection. After PS weighting, all patients treated with PBT were found to have superior median and 5 year survival than patients treated with XRT: 45.9 vs. 29.7 months (*p* = 0.009) and 46.1 vs. 35.5% (*p* = 0.0160), respectively.

**Conclusions:** PBT is associated with improved OS compared to XRT for patients with gliomas. This finding warrants verification in the randomized trial setting in order to account for potential patient imbalances not adequately captured by the NCDB, such as tumor molecular characteristics and patient performance status.

**Importance of the Study:** This is the first study that compares the outcomes of patients treated with photon based radiotherapy vs. proton based radiotherapy for patients with gliomas. In this retrospective analysis, the results demonstrate that proton therapy is associated with improved outcomes which support ongoing prospective, randomized clinical trials comparing the two modalities in patients with gliomas.

## Introduction

Approximately 20,000 adults are diagnosed with primary gliomas each year in the United States (1). The clinical outcomes are heterogeneous and largely depend on World Health Organization (WHO) histologic grade. For Grade I gliomas, the 5 year survival is estimated to be over 95% (2), whereas the median survival for Grade IV gliomas is often reported in months (3).

Advances in molecular genetics have allowed for the identification of additional prognostic and/or predictive mutations and epigenetic changes such as isocitrate dehydrogenase (IDH) mutation, chromosome 1p/19q co-deletion, and O (4)-methylguanine–DNA methyltransferase (MGMT) hypermethylation. These predictive biomarkers have helped us better define the role of adjuvant RT and chemotherapy for grade II-IV gliomas (4–6). For 1p/19q co-deleted or IDH-mutant Grade III glioma patients (7, 8), treatment with chemotherapy and radiation nearly doubles the median survival compared to radiation alone (6, 9). Similarly, the long-term results of RTOG 9802 demonstrated that the addition of chemotherapy to RT for grade II glioma patients improved median survival from 7.8 to 13.3 years (5). With patients living longer, the concern for long-term toxicity of therapy becomes increasingly important. In particular, RT can cause hypothalamic-pituitary-axis (HPA) dysfunction, neurocognitive changes, and an increased risk of developing secondary malignancy (10).

By virtue of the Bragg peak phenomenon, PBT differs from XRT in the use of charged particles, with a finite, energy-dependent range in tissue that can be adjusted to match the depth of the target (11). This is due to the fact that the energy lost by particulate radiation is inversely proportional to the square of their velocity—as an incident proton particle slows down, it deposits most of its energy prior to coming to a complete stop. This results in a steep dose fall-off at the end of the particle path allowing for better sparing of normal tissue. Clinically, this affords an opportunity to improve upon the therapeutic ratio of RT for primary gliomas through reducing or eliminating radiation exposure to non-target tissues. For gliomas, improved radiation avoidance of radiosensitive structures such as the hippocampus (12), cerebral cortex (13), HPA (14), and overall reduction in the volume of irradiated brain may improve upon quality of life endpoints including fatigue, neurocognitive dysfunction, and endocrine abnormalities.

The dosimetric advantages (15) of PBT and the safety of PBT for treatment of gliomas (16) have been previously reported. However, PBT is not as widely available as XRT, requires greater capital investment, is typically associated with greater costs, and has variable coverage by private insurance companies due to uncertainty about superiority of outcomes compared to XRT. To investigate the potential impact of PBT on overall survival (OS), our study utilized the large National Cancer Data Base (NCDB) to evaluate the clinical outcomes of patients with primary gliomas treated with XRT and PBT. We also sought to evaluate the practice patterns and usage trends for PBT in the United States.

## Methods

### Patient selection

The NCDB is maintained by the American College of Surgeons and the American Cancer Society and includes more than 1,500 Commission on Cancer (CoC)-approved hospitals in the United States. The 2014 Brain/CNS (Central Nervous System) NCDB Participant User File (PUF) was used to select patients for this study. This file includes patient demographics, socioeconomic factors, disease characteristics, treatment details and survival outcomes.

**Figure 1:**
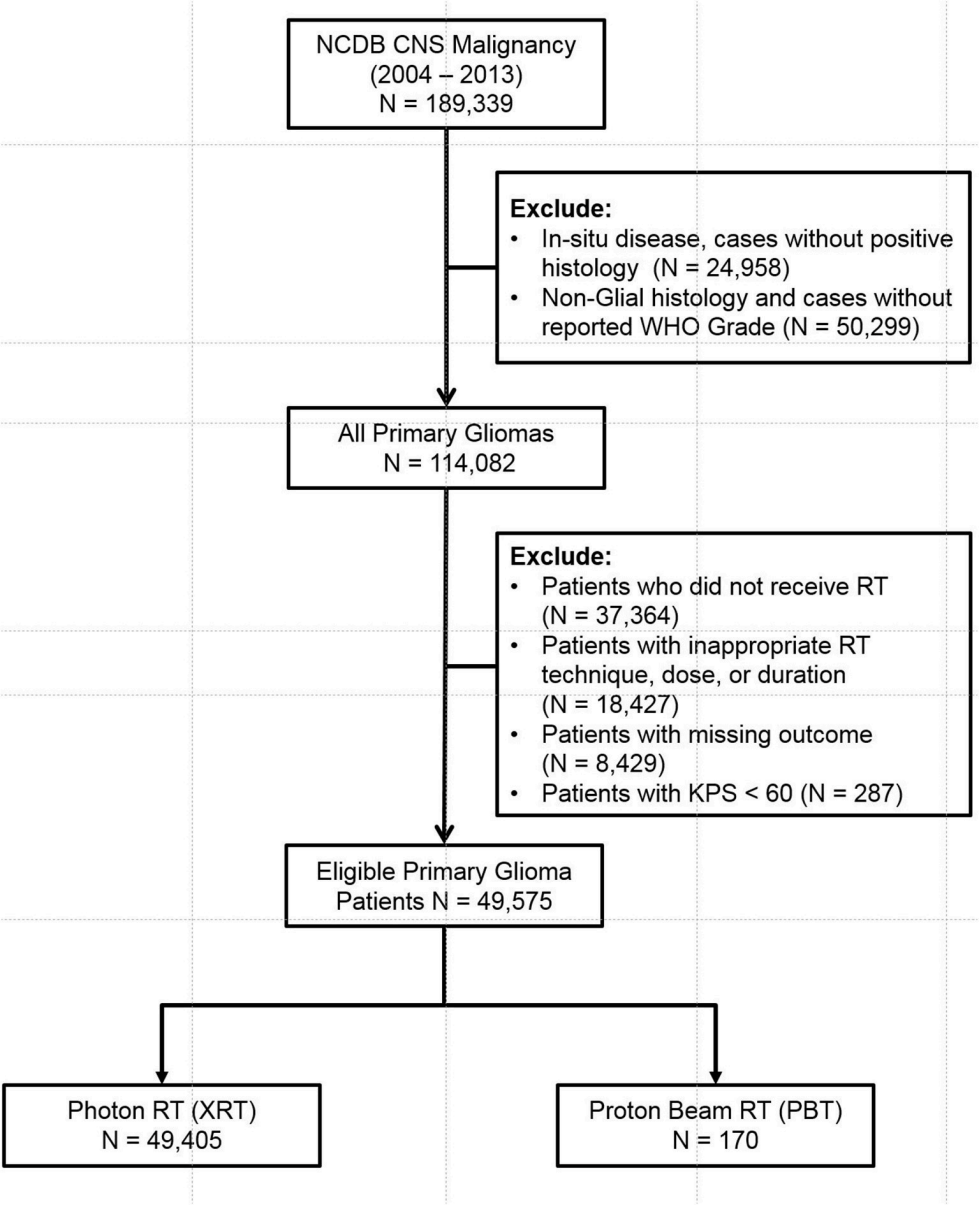
CONSORT Diagram for all patients.

The database was queried for patients diagnosed with CNS malignancy from 2004 to 2013. Adult patients (age > 18) with invasive, histologically confirmed, WHO Grade I-IV gliomas were included. Patients with non-glial histology (metastases, sarcoma, meningioma, hemangioma, embryonal tumors, ventricular tumors, and primitive neuroendocrine tumors) and patients who did not specifically receive RT to the brain were all excluded. Patients with Karnofsky Performance Status (KPS) of < 60% were also excluded. Patients who received inadequate RT dose (< 45 Gy), unconventional RT techniques (Cobalt, Electrons, Linac radiosurgery, Gamma Knife, Brachytherapy, Radium, and radioisotope), prolonged RT course (> 70 days), and cases with missing outcomes were also excluded. The eligible patients were then stratified into XRT and PBT groups (Figure 1, CONSORT Diagram for all patients).

### Patient demographics

The patient's age, gender, race, insurance status, median income quartile, urban/rural setting, treatment facility type (academic/research vs. community), treatment location (West, Northeast, etc.) great circle distance (distance in miles between patient's residence and the hospital that reported the case) were available for analysis. Note that treatment location pertains to geographic location within the continental United States of America. Charlson-Deyo Score was used as a surrogate for patient co-morbidities. Patient zip codes were used to determine urban vs. rural location. Metropolitan residence was defined as counties with population > 250,000. Rural (population < 2,500) and Urban patients (population > 2,500 but < 250,000) were combined into one group.

### Disease characteristics

The following tumor related variables were evaluated: year of diagnosis, primary site (frontal lobe, temporal lobe, etc.), laterality, focality (unifocal vs. multifocal), tumor size, WHO Grade, histology (e.g., astrocytoma, oligodendroglioma, glioblastoma), loss of heterozygosity (LOH) of chromosome 1p/19q. The patients were stratified into Group A: Low Grade Glioma (WHO Grade I & II) and Group B: High Grade Glioma (WHO Grade III & IV). Group A was then further stratified into oligodendroglioma, astrocytoma, and mixed histology. Group B was further stratified into anaplastic oligodendroglioma, anaplastic astrocytoma, mixed anaplastic oligoastrocytoma, and glioblastoma (GBM).

### Treatment characteristics

Radiation dose, radiation modality (PBT vs. XRT), use of chemotherapy, extent of surgery (gross total resection, subtotal resection, biopsy) were used for analysis. The XRT cohort was further sub-stratified into: Intensity Modulated Radiotherapy (IMRT), 3D-Conformal Radiotherapy (3DCRT), and Photon radiotherapy not otherwise specified (NOS). The NCDB defines 3DCRT as an external beam technique using multiple, fixed portals shaped to conform to a defined target volume. Photon RT NOS is defined as treatment is known to be by external beam, but with insufficient information provided to determine the specific modality.

### Outcome

OS was the primary outcome and was defined as time from diagnosis to time of death or last follow-up.

### Statistical analysis

The univariate association between each covariate and study cohorts were assessed using the χ^2^ test for categorical covariates and ANOVA for numerical covariates, and a multivariable (MVA) logistic regression was carried out for predicting utilization of PBT vs. XRT. The univariate association (UVA) between each covariate including study cohorts and study outcome was assessed using Cox proportional hazards models and log-rank tests. A multivariable Cox proportional hazard model was fit for OS. The MVA models were built by a backward variable selection method applying an α = 0.1 removal criterion. Kaplan-Meier (KM) plots were calculated to compare the survival curves by treatment cohorts.

We implemented a newly developed propensity score (PS) weighting schema in order to control any confounding effects due to baseline patient demographic, clinical, and treatment related differences (17). First, a logistic regression model was applied to estimate the probability of a patients could receive PBT based on his/her baseline characteristics as listed in Table 1, and this probability was defined as the propensity score (PS). Patients in PBT cohort were assigned a weight with value of 1-PS, while for patients in XRT cohort the weight was PS. The covariates balance between the two cohorts was evaluated by the standardized differences, and a value of < 0.2 was considered as negligible imbalance (18). The effects were estimated in the matched sample by a Cox model with a robust variance estimator for OS.

**Table 1:** Multivariable logistic regression for the receipt of proton vs. non-proton in all patients.

**Covariate**	**Level**	**Odds ratio (95% CI)**	**OR *P*-value**	**Type3 *P*-value**
Low/ High Grade Glioma	Group A: Low Grade Glioma	6.47 (4.05–10.34)	<**0.001**	<**0.001**
	Group B: High Grade Glioma	–	–
Facility Type	Academic/Research Program	2.99 (1.97–4.54)	<**0.001**	<**0.001**
	All others	1.25 (0.66–2.39)	0.494
	Unknown	–	–
Facility Location	Northeast	2.30 (1.30–4.09)	**0.004**	<**0.001**
	South	1.06 (0.55–2.06)	0.856
	West	5.52 (3.17–9.58)	<**0.001**
	Midwest	–	–
	Unknown	–	–
Urban/Rural 2003	Metro	2.71 (1.45–5.05)	**0.002**	**0.007**
	Unknown	2.58 (0.81–8.23)	0.110
	Urban + Rural	–	–
Surgery	Yes	1.76 (1.11–2.81)	**0.017**	**0.017**
	No	–	–
Age at Diagnosis		0.98 (0.96–0.99)	**0.006**	**0.006**

*The following variables were removed from the model: Charlson-Deyo Score, KPS and MGMT Combined, Focality, Insurance status, Income: Median Income Quartiles 2000, Race, Chemotherapy, Sex, Tumor size based on 6cm, and Radiation dose. Bold values reflect statistically significant variables with p-value < 0.05*.

## Results

Query of the 2014 NCDB Brain Participant User File (PUF) resulted in 189,339 cases. Patients with *in-situ* disease, non-glial histology, unavailable WHO Grade, and patients who did not receive RT were excluded. Patients with non-standard or missing RT technique or dose (< 45 Gy), prolonged RT course (> 70 days), and missing outcomes were further excluded. This yielded a total of 49,575 eligible patients (Figure 1). Patients were then stratified into two main groups—PBT (*n* = 170) and XRT (*n* = 49,405). The XRT cohort was further sub-stratified into 3D-CRT (*n* = 5,196), IMRT (*n* = 20,215), and Photon RT, NOS (*n* = 2,3994).

Supplementary Table 1 shows detailed patient demographics, disease characteristics, and treatment information. The median follow-up time for all patients was 62.1 months (62.3 months for XRT and 50.3 months for PBT). High Grade Glioma (HGG) represented 91.2% of all patients. The median age was 59 years for all patients. The median total RT dose for all patients was 60 Gy. Univariate analysis of all variables for XRT vs. PBT is shown in Supplementary Table 2.

### Variables associated with receipt of proton therapy

Table 1 illustrates the demographic, clinical, and treatment variables related to the use of PBT, with associated odds ratios (OR) and 95% confidence intervals (CI). MVA logistic regression model demonstrated multiple factors associated with increased likelihood of treatment with PBT: LGG (OR 6.47, CI [4.05-10.34], p < 0.001), treatment at academic facility [OR 2.99, CI [1.97–4.54], *p* < 0.001], west geographic location [OR 5.52, [CI 3.17–9.58], *p* < 0.001], surgical treatment [OR 1.76, CI [1.11–2.81], *p* = 0.017], and younger age [OR 0.98, CI [0.96–0.99], *p* = 0.006].

### Overall survival

UVA for OS are shown in Supplementary Table 3. PBT was associated with improved OS when compared to XRT [HR 0.47, CI [0.38–0.58], *p* < 0.001]. Evaluating the impact of PBT against the sub-stratification of XRT demonstrated that the association of increased OS with PBT persisted [HR 0.46, CI [0.37–0.57], *p* < 0.001] when compared with 3D-CRT and IMRT.

These results were then confirmed with MVA for OS, shown in Table 2a. PBT remained a significant factor associated with increased OS compared to XRT [HR 0.66, CI [0.53–0.83], *p* < 0.001]. PBT also predicted for higher OS [HR 0.66, CI [0.53–0.82], *p* < 0.001] when compared to 3D-CRT, IMRT and Photon RT NOS. Unadjusted Kaplan-Meier survival analysis for proton vs. photon radiotherapy is shown is supplementary Figure 1.

**Table 2a:** Multivariable analysis for overall survival.

**Covariate**	**Level**	**N**	**Hazard ratio (95% CI)**	**HR *P*-value**	**Type3 *P*-value**
Radiation Modality	Proton	159	0.66 (0.53–0.83)	<**0.001**	<**0.001**
	Non-Proton (XRT)	45888	–	–
Radiation Modality	3D-CRT IMRT Proton Photon-NOS	4304 18372 159 22155	1.00 (0.97–1.04) 0.97 (0.95–1.00) 0.66 (0.53–0.82) –	0.848 **0.022** <**0.001** –	<**0.001**

*The following variables were removed from the model: Grade, Urban/Rural 2003, and Radiation dose. Bold values reflect statistically significant variables with p-value < 0.05*.

### Sub-group survival analysis of HGG & LGG patients

For patients with LGG, PBT was a significant predictor on MVA for lower risk of death [HR 0.46, CI [0.22–0.98], *p* = 0.043] compared to XRT (Table 2b). For HGG patients, PBT also predicted for improved OS [HR 0.67, CI [0.53–0.84], *p* < 0.001], although the HR was lower than for LGG patients (Table 2b). PBT continued to be a predictor for OS when compared to IMRT for the HGG subgroup [HR 0.68, CI [0.54–0.86], *p* = 0.001].

**Table 2b:** Multivariable analysis for overall survival stratified by WHO Grade and XRT subgroups.

**Covariate**	**Level**	**N**	**Hazard ratio (95% CI)**	**HR *P*-value**
A: Low Grade Glioma	Proton vs. Non-Proton (XRT)	44 vs. 4102	0.46 (0.22–0.98)	**0.043**
B: High Grade Glioma	Proton vs. Non-Proton (XRT)	119 vs. 43106	0.67 (0.53–0.84)	<**0.001**
A: Low Grade Glioma	Proton vs. IMRT	44 vs. 1596	0.45 (0.24–1.05)	0.067
	Photon (NOS) vs. IMRT	2048 vs. 1596	1.13 (1.02–1.25)	**0.018**
	3D–CRT vs. IMRT	458 vs. 1596	1.04 (0.89–1.21)	0.644
B: High Grade Glioma	Proton vs. IMRT	119 vs. 17770	0.68 (0.54–0.86)	**0.001**
	Photon (NOS) vs. IMRT	20836 vs. 17770	1.04 (1.01–1.06)	**0.003**
	3D–CRT vs. IMRT	4500 vs. 17770	1.05 (1.01–1.09)	**0.009**

*Bold values reflect statistically significant variables with p-value < 0.05*.

### Propensity score analysis

**Figure 2:**
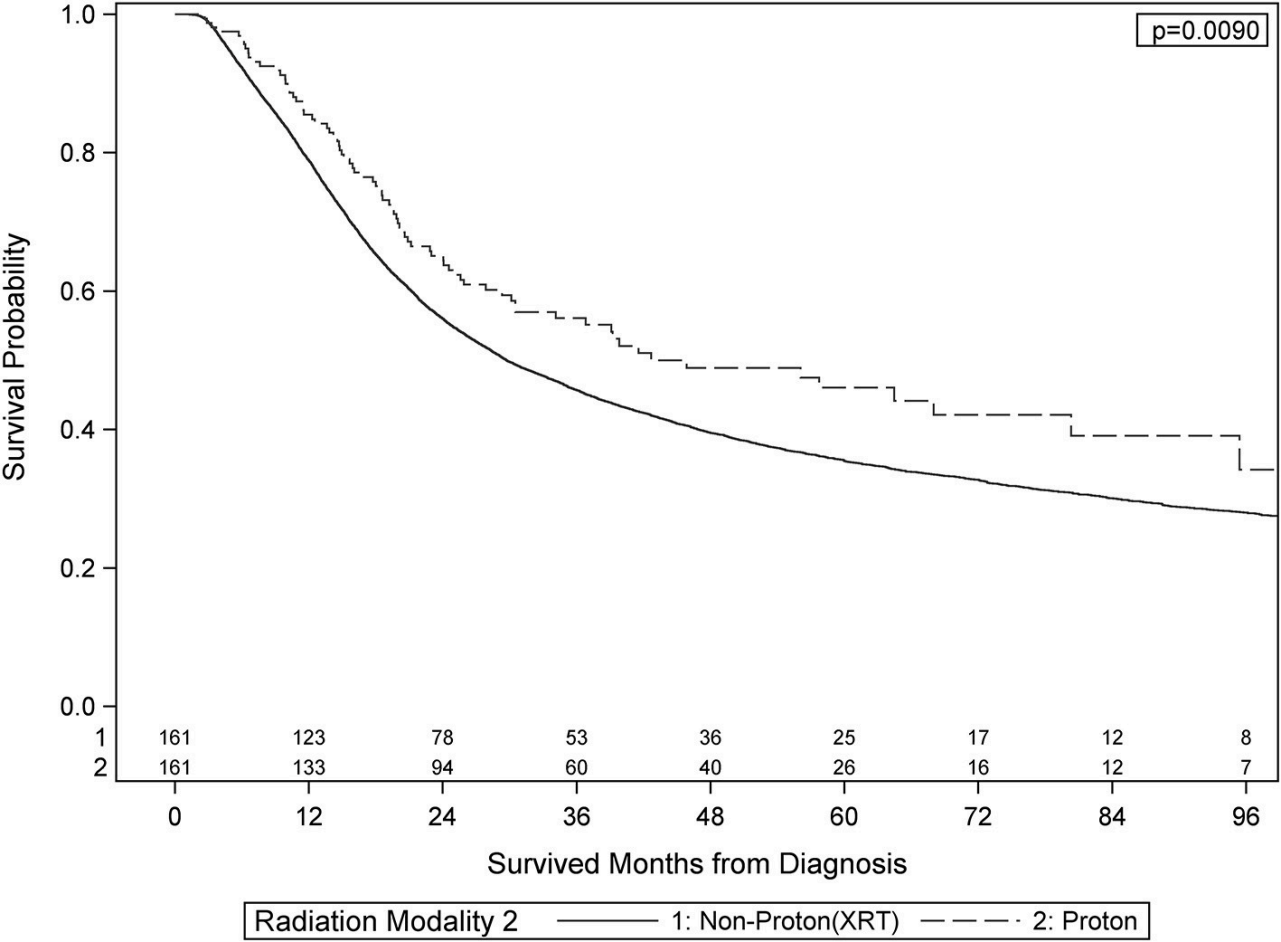
Adjusted Kaplan-Meier Survival Plot stratified by Proton vs. Non-Proton.

**Figure 3:**
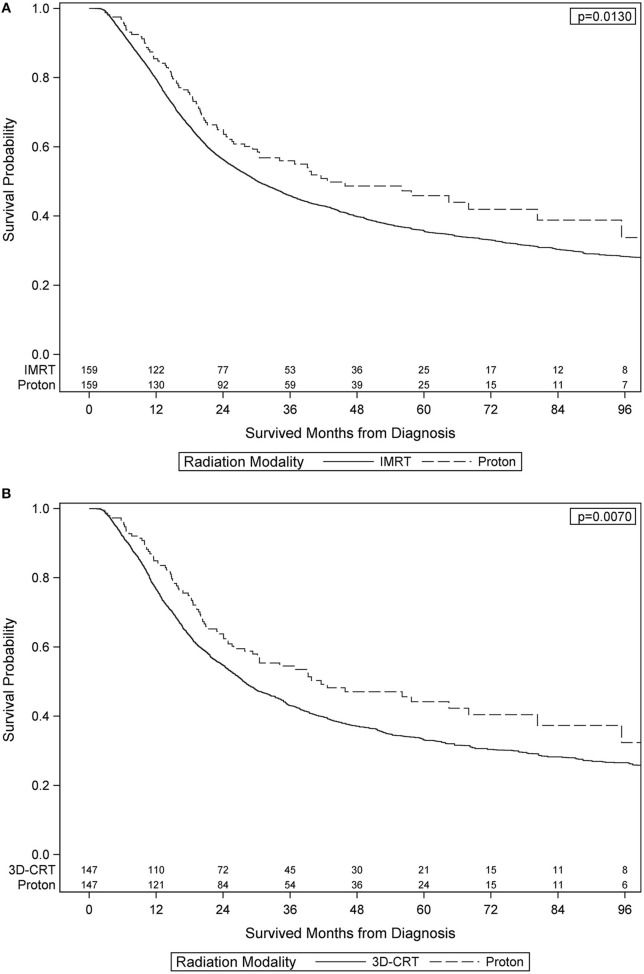
Adjusted Kaplan-Meier Plot stratified by Proton vs. IMRT Adjusted Kaplan-Meier Plot stratified by Proton vs. 3DCRT.

After PS weighting, the baseline patient demographics, disease characteristics, and treatment specifics were all similar between PBT and XRT cohorts (Table 3). Figure 2 (Adjusted KM Plot stratified by Proton vs. Non-Proton) shows the KM plots for the PS matched cohorts, stratified by PBT vs. XRT. PBT had higher median survival (45.9 vs. 29.7 months) and 5-year survival (46.1 vs. 35.5%; *p* = 0.009). Further PS adjusted analysis of the sub-stratification of the XRT group into IMRT and 3DCRT is shown in Figure 3A (Adjusted KM Plot stratified by Proton vs. IMRT) and Figure 3B (Adjusted KM Plot stratified by Proton vs. 3DCRT), respectively. After PS weighting, patients receiving PBT had statistically significant improved OS when compared to IMRT and 3DCRT (*p* < 0.05).

**Table 3:** Propensity score weighted baseline patient characteristics.

			**Radiation modality**			
**Covariate**	**Level**	**Statistics**	**Non-proton (XRT) *N* = 161**	**Proton (PBT) *N* = 161**	Parametric *P*-value^*^	**Standardized difference**
Low/ High Grade Glioma	Group A: Low Grade Glioma	N (Col%)	43 (26.69)	43 (26.69)	1.000	0.000
	Group B: High Grade Glioma	N (Col%)	118 (73.31)	118 (73.31)		0.000
Sex	Male	N (Col%)	96 (59.57)	96 (59.57)	1.000	0.000
	Female	N (Col%)	65 (40.43)	65 (40.43)		0.000
Income: Median Income Quartiles 2000	< $30,000	N (Col%)	16 (10.47)	16 (10.47)	1.000	0.000
	$30,000 – $35,999	N (Col%)	19 (12.34)	19 (12.34)		0.000
	$36,000 – $45,999	N (Col%)	45 (28.2)	45 (28.2)		0.000
	$46,000 +	N (Col%)	79 (48.99)	79 (48.99)		0.000
Facility Type	Academic/Research Program	N (Col%)	84 (52.11)	84 (52.11)	1.000	0.000
	All others	N (Col%)	32 (20.38)	32 (20.38)		0.000
	Unknown	N (Col%)	44 (27.5)	44 (27.5)		0.000
Facility Location	Northeast	N (Col%)	34 (21.38)	34 (21.38)	1.000	0.000
	South	N (Col%)	17 (11.13)	17 (11.13)		0.000
	Midwest	N (Col%)	17 (11.14)	17 (11.14)		0.000
	West	N (Col%)	46 (28.85)	46 (28.85)		0.000
	Unknown	N (Col%)	44 (27.5)	44 (27.5)		0.000
Year of Diagnosis	2004–2005	N (Col%)	20 (12.9)	20 (12.9)	1.000	0.000
	2006–2007	N (Col%)	19 (12.31)	19 (12.31)		0.000
	2008–2009	N (Col%)	24 (15.41)	24 (15.41)		0.000
	2010–2011	N (Col%)	40 (25.11)	40 (25.11)		0.000
	2012–2013	N (Col%)	55 (34.26)	55 (34.26)		0.000
Charlson-Deyo Score	0	N (Col%)	139 (86.45)	139 (86.45)	1.000	0.000
	1/ 2+	N (Col%)	21 (13.55)	21 (13.55)		0.000
Surgery	No	N (Col%)	20 (12.92)	20 (12.92)	1.000	0.000
	Yes	N (Col%)	140 (87.08)	140 (87.08)		0.000
KPS and MGMT Combined	Positive	N (Col%)	8 (5.49)	8 (5.49)	1.000	0.000
	Negative	N (Col%)	11 (7.3)	11 (7.3)		0.000
	Unknown	N (Col%)	140 (87.21)	140 (87.21)		0.000
Focality	Unifocal	N (Col%)	79 (49.57)	79 (49.57)	1.000	0.000
	Multifocal	N (Col%)	9 (6.12)	9 (6.12)		0.000
	Unknown	N (Col%)	71 (44.31)	71 (44.31)		0.000
Chemotherapy	No	N (Col%)	34 (21.35)	34 (21.35)	1.000	0.000
	Chemotherapy administered, type and number of agents not documented	N (Col%)	7 (4.89)	7 (4.89)		0.000
	Single-agent chemotherapy	N (Col%)	110 (68.27)	110 (68.27)		0.000
	Multiagent chemotherapy	N (Col%)	4 (3.09)	4 (3.09)		0.000
	Unknown	N (Col%)	3 (2.41)	3 (2.41)		0.000
Radiation dose	2: 4500–6000	N (Col%)	139 (86.48)	139 (86.48)	1.000	0.000
	3:> 6000	N (Col%)	21 (13.52)	21 (13.52)		0.000
Insurance status	Not Insured/Unknown	N (Col%)	6 (4.3)	6 (4.3)	1.000	0.000
	Private	N (Col%)	107 (66.81)	107 (66.81)		0.000
	Medicaid	N (Col%)	14 (9.19)	14 (9.19)		0.000
	Medicare/Other Government	N (Col%)	31 (19.7)	31 (19.7)		0.000
Tumor size based on 6cm	< 6cm	N (Col%)	93 (57.77)	93 (57.77)	1.000	0.000
	≥ 6cm	N (Col%)	27 (17.19)	27 (17.19)		0.000
	Unknown	N (Col%)	40 (25.04)	40 (25.04)		0.000
Age at Diagnosis		Mean (Std)	49.4 (0.88)	49.4 (14.51)	0.999	0.000

* *The parametric p-value is calculated by ANOVA for numerical covariates and Chi-Square test for categorical covariates*.

## Discussion

The ability to minimize radiation dose to normal tissue has evolved over time. From using plain films to treat entire lobes or hemispheres of the brain, to the adoption of computed tomography (CT) for more accurate delineation of target volumes, and finally to the current standard of IMRT technique where modification of the photon fluence allows for superior dose conformity and sparing of normal tissue. PBT, an advanced RT modality, represents another step in this evolution of maximizing conformity of the dose to the target and minimizing dose to adjacent normal tissue. While the dosimetric superiority of PBT for the treatment of gliomas have been reported previously (15), whether these dosimetric gains translate into a clinically meaningful reduction in toxicity or a potential survival benefit remains unproven.

This study was designed to investigate the hypothesis that PBT is associated with improved survival compared to XRT. Our results suggest that adults with gliomas treated with PBT have statistically significant superior OS than similar patients treated with XRT. PBT was also associated with higher survival when independently compared to IMRT and 3DCRT. This effect persisted for both HGG and LGG after propensity score matching to minimize the impact of selection bias. We also found that patients with younger age, LGG, academic treatment centers, metropolitan residence, west geographic location, and patients treated with surgery were more likely to receive PBT.

To the best of our knowledge, this is the first study that compares survival outcomes of adult glioma patients treated with PBT vs. XRT. These data, although retrospective and encumbered by the inherent limitations of a large national database, provides preliminary evidence for a potential clinical benefit of PBT in adult glioma. In the present study, patients with LGG derived a higher magnitude of survival benefit with PBT than did patients with HGG. Since LGG typically affects young adults, it is possible that the impact of PBT appears greater in this setting due to its ability to spare the late toxicities associated with non-proton RT.

For HGG, the benefit of reducing late toxicities is limited by the relatively modest survival of these patients. However, the ability to spare radiosensitive normal tissues—circulating CD4+ lymphocytes—with the favorable dose profile of PBT has the potential to improve survival by an underlying immune mechanism, as emerging data indicates (19, 20). To that end, there have been prior Phase I/II studies that have utilized PBT for dose escalation in patients with GBM in the pre-temozolomide era which have resulted in modest improvement in survival in the single institutional setting (21, 22). At the time of submission of this manuscript, NRG-BN001 is ongoing—an open randomized, Phase II, multi-institutional trial comparing dose-escalated photon IMRT or PBT vs. conventional photon irradiation with concurrent and adjuvant temozolomide in patients with newly diagnosed GBM (23).

The other possibility that should be considered is that proton therapy could potentially be associated with increased toxicity. Current data suggests that at the distal end of the Bragg Peak, which, clinically is located at the tumor normal tissue interface, the relative biological effectiveness (RBE) and linear energy transfer (LTE) values increase exponentially. If the distal end of the Bragg peak is located adjacent to the amygdala or hippocampus, this could lead potentially higher rates of neurocognitive side effects as demonstrated in the early results of a single institutional Phase II randomized trial of proton vs. photon therapy for GBM. Nonetheless, long term results of this study are eagerly awaited.

Although there have been prospective studies investigating of the safety of PBT and progression free survival (PFS) in LGG (24), the effect of PBT on OS has yet to be reported. Wilkinson et al. (16) reported, in abstract form, acute toxicity results from the Proton Collaborative Group study for patients with LGG. OS was again not included in that report. A report of neurocognitive outcomes in patients treated with PBT for LGG has also been published, with promising preservation of cognitive functioning (25). Building upon these findings, the NRG oncology group has proposed a phase III randomized study, NRG BN005, comparing PBT to photon radiation in patients with Grade II or grade III gliomas (26). Our study helps to further support the rationale for this initiative.

Although the findings that PBT is associated with improved survival is provocative, the survival benefit maybe due to selection bias. Patients seeking PBT often have additional means, including better access to clinical trial enrollment and successful salvage therapies, which may contribute to their improved OS. Although MVA and propensity score matching help to address known differences in groups, it cannot address variables not captured in the NCDB. The present study has a few other notable limitations. Due to the small number of patients in the PBT group and missing molecular characteristics, sub-group analysis for MGMT methylated, IDH mutated, and 1p/19 co-deleted tumors could not be performed. Moreover, we were unable to report on acute and late toxicities for patients since this information is not available in the NCDB. Lastly, performance status was not available for patients and this is an established prognostic factor for gliomas (27, 28). Such variables not adequately recorded in the NCDB serve as an inherent limitation for large database studies. With limited information available for important prognostic variables such as performance status, MGMT methylation, and 1p19q co-deletion, the results of our study will require validation in a randomized clinical trial setting where such variables are adequately recorded.

## Conclusions

In this NCDB analysis, compared to XRT, PBT was associated with improved OS in adult patients with LGG and HGG. Although the retrospective nature and inability to account for all potential confounding factors limit definitive conclusions, these data are hypothesis-generating and support ongoing prospective, randomized clinical trials comparing PBT to XRT in LGG and HGG patients.

## Ethics statement

This study utilized the National Cancer Data Base (NCDB) which is a multi-institutional, de-identified cancer registry. Therefore, informed consent or ethics approval is not applicable.

## Author contributions

All authors listed have made a substantial, direct and intellectual contribution to the work, and approved it for publication.

### Conflict of interest statement

The authors declare that the research was conducted in the absence of any commercial or financial relationships that could be construed as a potential conflict of interest.

## Supplementary material

Supplementary Figure 1 Unadjusted KM Plot for Proton vs. XRT.

Supplementary Table 1 Baseline Characteristics of all patients.

Supplementary Table 2 Baseline Characteristics Stratified by Proton vs. XRT.

Supplementary Table 3 Univariate Analysis for OS.
