# Supplementary figures and images for: Proton vs. Photon Radiation Therapy for Primary Gliomas: An Analysis of the National Cancer Data Base
Source: Front Oncol. 2018 Nov 28;8:440. doi: 10.3389/fonc.2018.00440 (PMC6279888; doi:10.3389/fonc.2018.00440)

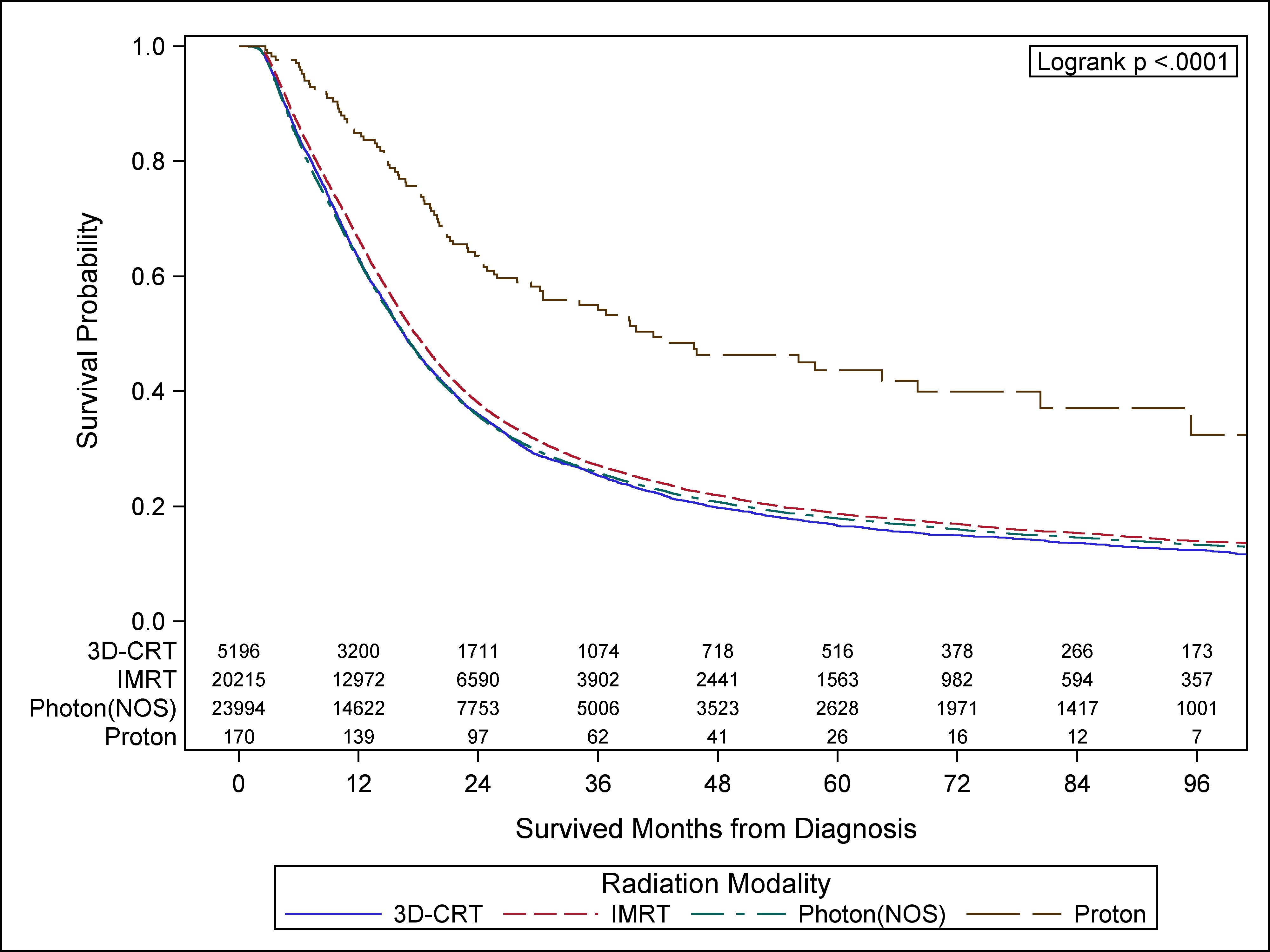

Supplement: Supplementary Figure 1 — Unadjusted KM Plot for Proton vs. XRT. [file Image_1.JPEG]
