# Supplementary material for: Proton vs. Photon Radiation Therapy for Primary Gliomas: An Analysis of the National Cancer Data Base
Source: Front Oncol. 2018 Nov 28;8:440. doi: 10.3389/fonc.2018.00440 (PMC6279888; doi:10.3389/fonc.2018.00440)
Supplement: Supplementary Table 1 — Baseline Characteristics of all patients. [file Data_Sheet_2.doc]

Variable	Level	N (%) = 49575	
Radiation Modality	3D-CRT	5196 (10.5)	
	IMRT	20215 (40.8)	
	Photon(NOS)	23994 (48.4)	
	Proton	170 (0.3)	
	
Radiation Modality 2	Non-Proton(XRT)	49405 (99.7)	
	Proton	170 (0.3)	
	
Age Category Based on 40	Age < 40	5638 (11.4)	
	Age >= 40	43937 (88.6)	
	
Sex	Male	29041 (58.6)	
	Female	20534 (41.4)	
	
Race	White	45143 (91.1)	
	Black	2670 (5.4)	
	Others/Unknown	1762 (3.6)	
	
Education: Percent No High School Degree Quartiles 2000	Not Available	2204	
	>=29%	6019 (12.7)	
	20-28.9%	10134 (21.4)	
	14-19.9%	11555 (24.4)	
	< 14%	19663 (41.5)	
	
Income: Median Income Quartiles 2000	Not Available	2194	
	< $30,000	4646 (9.8)	
	$30,000 - $35,999	7900 (16.7)	
	$36,000 - $45,999	13409 (28.3)	
	$46,000 +	21426 (45.2)	
	
Facility Type	Academic/Research Program	20606 (41.6)	
	All others	23331 (47.1)	
	Unknown	5638 (11.4)	
	
Facility Location	Northeast	9330 (18.8)	
	South	13945 (28.1)	
	Midwest	13006 (26.2)	
	West	7656 (15.4)	
	Unknown	5638 (11.4)	
	
Urban/Rural 2003	Metro	38484 (77.6)	
	Urban + Rural	8979 (18.1)	
	Unknown	2112 (4.3)	
	
Insurance status	Not Insured/Unknown	2715 (5.5)	
	Private	27781 (56.0)	
	Medicaid	3391 (6.8)	
	Medicare/Other Government	15688 (31.6)	
	
Year of Diagnosis	2004-2005	7309 (14.7)	
	2006-2007	8564 (17.3)	
	2008-2009	9982 (20.1)	
	2010-2011	11545 (23.3)	
	2012-2013	12175 (24.6)	
	
Grade	Poorly Differentiated/Undifferentiated	23260 (46.9)	
	Cell Type Not Determined	24304 (49.0)	
	Well/ Moderately Differentiated	2011 (4.1)	
	
Charlson-Deyo Score	0	38558 (77.8)	
	1/ 2+	11017 (22.2)	
	
Surgery	No	10027 (20.2)	
	Yes	39548 (79.8)	
	
Chromosome 19q: Loss of Heterozygosity	Positive	775 (1.6)	
	Negative	1512 (3.0)	
	Unknown	47288 (95.4)	
	
Chromosome 1p: Loss of Heterozygosity	Positive	716 (1.4)	
	Negative	1522 (3.1)	
	Unknown	47337 (95.5)	
	
Functional Neurologic Status - Karnofsky Performance Scale (KPS)	60	242 (0.5)	
	70	577 (1.2)	
	80	861 (1.7)	
	90	1075 (2.2)	
	100	326 (0.7)	
	888	2204 (4.4)	
	988	24810 (50.0)	
	999	19480 (39.3)	
	
Methylation of O6-Methylguanine-Methyltransferase (MGMT)	10	1251 (2.5)	
	20	1895 (3.8)	
	888	2204 (4.4)	
	988	23357 (47.1)	
	998	3949 (8.0)	
	999	16919 (34.1)	
	
Focality	Unifocal	17906 (36.1)	
	Multifocal	3108 (6.3)	
	Unknown	28561 (57.6)	
	
Surgery Extent	Gross total resection	6066 (12.2)	
	Subtotal resection	5911 (11.9)	
	Biopsy	4856 (9.8)	
	Others	27251 (55.0)	
	Unknown	5491 (11.1)	
	
Chemotherapy	No	6466 (13.0)	
	Chemotherapy administered, type and number of agents not documented	1708 (3.4)	
	Single-agent chemotherapy	37229 (75.1)	
	Multiagent chemotherapy	3723 (7.5)	
	Unknown	449 (0.9)	
	
Radiation dose	2: 4500 - 6000	42728 (86.2)	
	3:> 6000	6847 (13.8)	
	
Surgical Procedure of Primary Site at any CoC Facility	No	10012 (20.2)	
	Yes	39548 (79.8)	
	Unknown	15 (0.0)	
	
Great Circle Distance (quartile)	>=0, <=6	12252 (24.7)	
	>6, <=13	12036 (24.3)	
	>13, <=30	12097 (24.4)	
	>30, <=3942	12108 (24.4)	
	Unknown	1082 (2.2)	
	
Tumor size based on 6cm	< 6cm	30535 (61.6)	
	>= 6cm	7722 (15.6)	
	Unknown	11318 (22.8)	
	
Low/ High Grade Glioma	Group A: Low Grade Glioma	4351 (8.8)	
	Group B: High Grade Glioma	45224 (91.2)	
	
Low/ High Grade Glioma + Histology	Group A - Oligodendroglioma	1017 (2.1)	
	Group A - Astrocytoma	2007 (4.2)	
	Group A - Other	882 (1.8)	
	Group B - Oligodendroglioma	1692 (3.5)	
	Group B - Astrocytoma	6759 (14.0)	
	Group B - Glioblastoma	33931 (70.4)	
	Group B - Other	1906 (4.0)	
	Missing	1381	
	
KPS and MGMT Combined	Positive	897 (1.8)	
	Negative	1363 (2.7)	
	Unknown	47315 (95.4)	
	
Age at Diagnosis	Mean	57.30	
	Median	59.00	
	Minimum	18.00	
	Maximum	90.00	
	Std Dev	13.96	
	Missing	0.00	
	
Great Circle Distance	Mean	37.42	
	Median	12.50	
	Minimum	0.00	
	Maximum	3941.50	
	Std Dev	128.61	
	Missing	1082.00	
	
Tumor Size (cm)	Mean	4.85	
	Median	4.30	
	Minimum	0.10	
	Maximum	98.90	
	Std Dev	6.59	
	Missing	11318.00	
	
Note that values of 888, 988, and 998, and 999 represent missing or unavailable data.
