# Supplementary material for: Proton vs. Photon Radiation Therapy for Primary Gliomas: An Analysis of the National Cancer Data Base
Source: Front Oncol. 2018 Nov 28;8:440. doi: 10.3389/fonc.2018.00440 (PMC6279888; doi:10.3389/fonc.2018.00440)
Supplement: Supplementary Table 2 — Baseline Characteristics Stratified by Proton vs. XRT. [file Data_Sheet_3.doc]

	Radiation Modality		
	___________________________		
Covariate	Statistics	Level	Non-Proton(XRT) N=49405	Proton N=170	Parametric P-value*	
Age Category Based on 40	N (Col %)	Age < 40	5592 (11.32)	46 (27.06)	<.001	
	N (Col %)	Age >= 40	43813 (88.68)	124 (72.94)		
	
Sex	N (Col %)	Male	28940 (58.58)	101 (59.41)	0.825	
	N (Col %)	Female	20465 (41.42)	69 (40.59)		
	
Race	N (Col %)	White	44992 (91.07)	151 (88.82)	0.039	
	N (Col %)	Black	2663 (5.39)	7 (4.12)		
	N (Col %)	Others/Unknown	1750 (3.54)	12 (7.06)		
	
Education: Percent No High School Degree Quartiles 2000	N (Col %)	>=29%	6002 (12.71)	17 (10.43)	0.522	
	N (Col %)	20-28.9%	10092 (21.38)	42 (25.77)		
	N (Col %)	14-19.9%	11516 (24.39)	39 (23.93)		
	N (Col %)	< 14%	19598 (41.51)	65 (39.88)		
	
Income: Median Income Quartiles 2000	N (Col %)	< $30,000	4629 (9.8)	17 (10.43)	0.474	
	N (Col %)	$30,000 - $35,999	7880 (16.69)	20 (12.27)		
	N (Col %)	$36,000 - $45,999	13363 (28.3)	46 (28.22)		
	N (Col %)	$46,000 +	21346 (45.21)	80 (49.08)		
	
Facility Type	N (Col %)	Academic/Research Program	20516 (41.53)	90 (52.94)	<.001	
	N (Col %)	All others	23297 (47.16)	34 (20)		
	N (Col %)	Unknown	5592 (11.32)	46 (27.06)		
	
Facility Location	N (Col %)	Northeast	9295 (18.81)	35 (20.59)	<.001	
	N (Col %)	South	13926 (28.19)	19 (11.18)		
	N (Col %)	Midwest	12988 (26.29)	18 (10.59)		
	N (Col %)	West	7604 (15.39)	52 (30.59)		
	N (Col %)	Unknown	5592 (11.32)	46 (27.06)		
	
Urban/Rural 2003	N (Col %)	Metro	38333 (77.59)	151 (88.82)	<.001	
	N (Col %)	Urban + Rural	8968 (18.15)	11 (6.47)		
	N (Col %)	Unknown	2104 (4.26)	8 (4.71)		
	
Insurance status	N (Col %)	Not Insured/Unknown	2708 (5.48)	7 (4.12)	0.006	
	N (Col %)	Private	27667 (56)	114 (67.06)		
	N (Col %)	Medicaid	3376 (6.83)	15 (8.82)		
	N (Col %)	Medicare/Other Government	15654 (31.69)	34 (20)		
	
Year of Diagnosis	N (Col %)	2004-2005	7284 (14.74)	25 (14.71)	0.065	
	N (Col %)	2006-2007	8543 (17.29)	21 (12.35)		
	N (Col %)	2008-2009	9955 (20.15)	27 (15.88)		
	N (Col %)	2010-2011	11504 (23.29)	41 (24.12)		
	N (Col %)	2012-2013	12119 (24.53)	56 (32.94)		
	
Grade	N (Col %)	Poorly Differentiated/Undifferentiated	23168 (46.89)	92 (54.12)	0.001	
	N (Col %)	Cell Type Not Determined	24240 (49.06)	64 (37.65)		
	N (Col %)	Well/ Moderately Differentiated	1997 (4.04)	14 (8.24)		
	
Charlson-Deyo Score	N (Col %)	0	38410 (77.75)	148 (87.06)	0.004	
	N (Col %)	1/ 2+	10995 (22.25)	22 (12.94)		
	
Surgery	N (Col %)	No	10006 (20.25)	21 (12.35)	0.010	
	N (Col %)	Yes	39399 (79.75)	149 (87.65)		
	
Chromosome 19q: Loss of Heterozygosity	N (Col %)	Positive	766 (1.55)	9 (5.29)	<.001	
	N (Col %)	Negative	1500 (3.04)	12 (7.06)		
	N (Col %)	Unknown	47139 (95.41)	149 (87.65)		
	
Chromosome 1p: Loss of Heterozygosity	N (Col %)	Positive	708 (1.43)	8 (4.71)	<.001	
	N (Col %)	Negative	1509 (3.05)	13 (7.65)		
	N (Col %)	Unknown	47188 (95.51)	149 (87.65)		
	
Functional Neurologic Status - Karnofsky Performance Scale (KPS)	N (Col %)	60	242 (0.49)	0 (0)	<.001	
	N (Col %)	70	574 (1.16)	3 (1.76)		
	N (Col %)	80	860 (1.74)	1 (0.59)		
	N (Col %)	90	1074 (2.17)	1 (0.59)		
	N (Col %)	100	319 (0.65)	7 (4.12)		
	N (Col %)	888	2203 (4.46)	1 (0.59)		
	N (Col %)	988	24739 (50.07)	71 (41.76)		
	N (Col %)	999	19394 (39.26)	86 (50.59)		
	
Methylation of O6-Methylguanine-Methyltransferase (MGMT)	N (Col %)	10	1249 (2.53)	2 (1.18)	0.001	
	N (Col %)	20	1888 (3.82)	7 (4.12)		
	N (Col %)	888	2203 (4.46)	1 (0.59)		
	N (Col %)	988	23289 (47.14)	68 (40)		
	N (Col %)	998	3924 (7.94)	25 (14.71)		
	N (Col %)	999	16852 (34.11)	67 (39.41)		
	
Focality	N (Col %)	Unifocal	17825 (36.08)	81 (47.65)	0.007	
	N (Col %)	Multifocal	3098 (6.27)	10 (5.88)		
	N (Col %)	Unknown	28482 (57.65)	79 (46.47)		
	
Surgery Extent	N (Col %)	Gross total resection	6037 (12.22)	29 (17.06)	<.001	
	N (Col %)	Subtotal resection	5877 (11.9)	34 (20)		
	N (Col %)	Biopsy	4839 (9.79)	17 (10)		
	N (Col %)	Others	27171 (55)	80 (47.06)		
	N (Col %)	Unknown	5481 (11.09)	10 (5.88)		
	
Chemotherapy	N (Col %)	No	6429 (13.01)	37 (21.76)	<.001	
	N (Col %)	Chemotherapy administered, type and number of agents not documented	1700 (3.44)	8 (4.71)		
	N (Col %)	Single-agent chemotherapy	37114 (75.12)	115 (67.65)		
	N (Col %)	Multiagent chemotherapy	3718 (7.53)	5 (2.94)		
	N (Col %)	Unknown	444 (0.9)	5 (2.94)		
	
Radiation dose	N (Col %)	2: 4500 - 6000	42581 (86.19)	147 (86.47)	0.915	
	N (Col %)	3:> 6000	6824 (13.81)	23 (13.53)		
	
Surgical Procedure of Primary Site at any CoC Facility	N (Col %)	No	9991 (20.22)	21 (12.35)	0.037	
	N (Col %)	Yes	39399 (79.75)	149 (87.65)		
	N (Col %)	Unknown	15 (0.03)	0 (0)		
	
Great Circle Distance (quartile)	N (Col %)	>=0, <=6	12231 (24.76)	21 (12.35)	<.001	
	N (Col %)	>6, <=13	12006 (24.3)	30 (17.65)		
	N (Col %)	>13, <=30	12041 (24.37)	56 (32.94)		
	N (Col %)	>30, <=3942	12050 (24.39)	58 (34.12)		
	N (Col %)	Unknown	1077 (2.18)	5 (2.94)		
	
Tumor size based on 6cm	N (Col %)	< 6cm	30437 (61.61)	98 (57.65)	0.570	
	N (Col %)	>= 6cm	7693 (15.57)	29 (17.06)		
	N (Col %)	Unknown	11275 (22.82)	43 (25.29)		
	
Low/ High Grade Glioma	N (Col %)	Group A: Low Grade Glioma	4305 (8.71)	46 (27.06)	<.001	
	N (Col %)	Group B: High Grade Glioma	45100 (91.29)	124 (72.94)		
	
Low/ High Grade Glioma + Histology	N (Col %)	Group A - Oligodendroglioma	999 (2.08)	18 (10.84)	<.001	
	N (Col %)	Group A - Astrocytoma	1986 (4.14)	21 (12.65)		
	N (Col %)	Group A - Other	876 (1.82)	6 (3.61)		
	N (Col %)	Group B - Oligodendroglioma	1684 (3.51)	8 (4.82)		
	N (Col %)	Group B - Astrocytoma	6729 (14.01)	30 (18.07)		
	N (Col %)	Group B - Glioblastoma	33868 (70.52)	63 (37.95)		
	N (Col %)	Group B - Other	1886 (3.93)	20 (12.05)		
	
KPS and MGMT Combined	N (Col %)	Positive	888 (1.8)	9 (5.29)	<.001	
	N (Col %)	Negative	1351 (2.73)	12 (7.06)		
	N (Col %)	Unknown	47166 (95.47)	149 (87.65)		
	
Age at Diagnosis	N		49405	170	<.001	
	Mean		57.33	49.42		
	Median		59	51		
	Min		18	18		
	Max		90	82		
	Std Dev		13.95	14.61		
	
Great Circle Distance	N		48328	165	0.004	
	Mean		37.32	65.84		
	Median		12.5	18.5		
	Min		0	0.4		
	Max		3941.5	1490.5		
	Std Dev		128.36	186.98		
	
Tumor Size (cm)	N		38130	127	0.587	
	Mean		4.84	5.16		
	Median		4.3	4.3		
	Min		0.1	0.1		
	Max		98.9	98.8		
	Std Dev		6.58	8.6		
	
Survived Months from Diagnosis	N		49405	170	<.001	
	Mean		24.55	35.41		
	Median		16.07	27.7		
	Min		0	2.69		
	Max		142.69	134.57		
	Std Dev		24.79	27.93		
	
*  The parametric p-value is calculated by ANOVA for numerical covariates and chi-square test for categorical covariates. 
Note that values of 888, 988, and 998, and 999 represent missing or unavailable data.	
