# Supplementary material for: Proton vs. Photon Radiation Therapy for Primary Gliomas: An Analysis of the National Cancer Data Base
Source: Front Oncol. 2018 Nov 28;8:440. doi: 10.3389/fonc.2018.00440 (PMC6279888; doi:10.3389/fonc.2018.00440)
Supplement: Supplementary Table 3 — Univariate Analysis for OS. [file Data_Sheet_4.doc]

		
	----------------------------------------	
Covariate	Level	N	Hazard Ratio (95% CI)	HR P-value	Log-rank P-value	
Treatment 	3D-CRT	4627	1.00 (0.96-1.03)	0.900	<.001	
	IMRT	19647	0.94 (0.92-0.96)	<.001		
	Proton	170	0.46 (0.37-0.57)	<.001		
	Photon-NOS	23994	-	-		
	
Radiation Modality 	Proton	170	0.47 (0.38-0.58)	<.001	<.001	
	Non-Proton(XRT)	49405	-	-		
	
Low/ High Grade Glioma	Group B: High Grade Glioma	45224	3.36 (3.21-3.52)	<.001	<.001	
	Group A: Low Grade Glioma	4351	-	-		
	
Low/ High Grade Glioma + Histology	Group A - Oligodendroglioma	1017	0.35 (0.31-0.40)	<.001	<.001	
	Group A - Astrocytoma	2007	0.86 (0.79-0.94)	<.001		
	Group A - Other	882	0.54 (0.48-0.61)	<.001		
	Group B - Oligodendroglioma	1692	0.64 (0.58-0.70)	<.001		
	Group B - Astrocytoma	6759	1.49 (1.39-1.59)	<.001		
	Group B - Glioblastoma	33931	3.05 (2.86-3.24)	<.001		
	Group B - Other	1906	-	-		
	
WHO Grade	Stage III	9417	1.74 (1.65-1.83)	<.001	<.001	
	Stage IV	35807	4.26 (4.07-4.47)	<.001		
	Stage I + II	4351	-	-		
	
Histology Category	Group III	2741	0.22 (0.20-0.23)	<.001	<.001	
	Group I + II	4689	0.51 (0.49-0.53)	<.001		
	Others	42145	-	-		
	
Age Category Based on 40	Age < 40	5638	0.28 (0.26-0.29)	<.001	<.001	
	Age >= 40	43937	-	-		
	
Sex	Male	29041	1.09 (1.07-1.11)	<.001	<.001	
	Female	20534	-	-		
	
Race	White	45143	1.33 (1.26-1.41)	<.001	<.001	
	Black	2670	1.22 (1.14-1.31)	<.001		
	Others/Unknown	1762	-	-		
	
Education: Percent No High School Degree Quartiles 2000	>=29%	6019	1.00 (0.96-1.03)	0.827	<.001	
	20-28.9%	10134	1.05 (1.02-1.07)	0.002		
	14-19.9%	11555	1.04 (1.02-1.07)	0.001		
	< 14%	19663	-	-		
	
Income: Median Income Quartiles 2000	< $30,000	4646	1.06 (1.03-1.10)	<.001	<.001	
	$30,000 - $35,999	7900	1.08 (1.05-1.11)	<.001		
	$36,000 - $45,999	13409	1.07 (1.05-1.10)	<.001		
	$46,000 +	21426	-	-		
	
Facility Type	Academic/Research Program	20606	3.35 (3.21-3.50)	<.001	<.001	
	All others	23331	3.90 (3.74-4.06)	<.001		
	Unknown	5638	-	-		
	
Facility Location	Northeast	9330	3.52 (3.36-3.68)	<.001	<.001	
	South	13945	3.77 (3.61-3.94)	<.001		
	Midwest	13006	3.68 (3.52-3.84)	<.001		
	West	7656	3.44 (3.28-3.61)	<.001		
	Unknown	5638	-	-		
	
Urban/Rural 2003	Metro	38484	0.93 (0.91-0.95)	<.001	<.001	
	Unknown	2112	1.14 (1.08-1.20)	<.001		
	Urban + Rural	8979	-	-		
	
Insurance status	Not Insured/Unknown	2715	1.03 (0.97-1.09)	0.352	<.001	
	Private	27781	1.10 (1.05-1.15)	<.001		
	Medicare/Other Government	15688	2.30 (2.20-2.40)	<.001		
	Medicaid	3391	-	-		
	
Year of Diagnosis	2004-2005	7309	0.97 (0.93-1.00)	0.045	<.001	
	2006-2007	8564	0.94 (0.91-0.97)	<.001		
	2008-2009	9982	0.96 (0.93-0.99)	0.018		
	2010-2011	11545	0.94 (0.92-0.97)	<.001		
	2012-2013	12175	-	-		
	
Grade	Poorly Differentiated/Undifferentiated	23260	2.68 (2.51-2.85)	<.001	<.001	
	Cell Type Not Determined	24304	2.99 (2.80-3.18)	<.001		
	Well/ Moderately Differentiated	2011	-	-		
	
Charlson-Deyo Score	0	38558	0.69 (0.68-0.71)	<.001	<.001	
	1/ 2+	11017	-	-		
	
Chromosome 19q: Loss of Heterozygosity	Negative	1512	2.30 (1.99-2.66)	<.001	<.001	
	Unknown	47288	3.76 (3.30-4.28)	<.001		
	Positive	775	-	-		
	
Chromosome 1p: Loss of Heterozygosity	Negative	1522	2.09 (1.80-2.42)	<.001	<.001	
	Unknown	47337	3.51 (3.09-4.00)	<.001		
	Positive	716	-	-		
	
KPS and MGMT Combined	Negative	1363	1.85 (1.62-2.11)	<.001	<.001	
	Unknown	47315	3.11 (2.78-3.47)	<.001		
	Positive	897	-	-		
	
Focality	Unifocal	17906	0.93 (0.91-0.95)	<.001	<.001	
	Multifocal	3108	1.59 (1.53-1.66)	<.001		
	Unknown	28561	-	-		
	
Surgery	Yes	39548	0.67 (0.66-0.69)	<.001	<.001	
	No	10027	-	-		
	
Radiation dose	2: 4500 - 6000	42728	0.87 (0.85-0.90)	<.001	<.001	
	3:> 6000	6847	-	-		
	
Tumor size based on 6cm	< 6cm	30535	1.16 (1.13-1.19)	<.001	<.001	
	>= 6cm	7722	0.93 (0.90-0.96)	<.001		
	Unknown	11318	-	-		
	
Great Circle Distance (quartile)	>=0, <=6	12252	0.69 (0.65-0.74)	<.001	<.001	
	>6, <=13	12036	0.66 (0.62-0.71)	<.001		
	>13, <=30	12097	0.64 (0.60-0.69)	<.001		
	>30, <=3942	12108	0.62 (0.58-0.67)	<.001		
	Unknown	1082	-	-		
	
Age at Diagnosis		49575	1.04 (1.04-1.05)	<.001	-	
	
Great Circle Distance		48493	1.00 (1.00-1.00)	<.001	-	
	
Tumor Size (cm)		38257	0.99 (0.99-1.00)	<.001	-	
	
